# Lysosomal accumulation of anticancer drugs triggers lysosomal exocytosis

**DOI:** 10.18632/oncotarget.15155

**Published:** 2017-02-07

**Authors:** Benny Zhitomirsky, Yehuda G. Assaraf

**Affiliations:** ^1^ The Fred Wyszkowski Cancer Research Laboratory, Department of Biology, Technion-Israel Institute of Technology, Haifa, Israel

**Keywords:** chemotherapeutics, multidrug resistance, lysosomes, drug sequestration, lysosomal exocytosis

## Abstract

We have recently shown that hydrophobic weak base anticancer drugs are highly sequestered in acidic lysosomes, inducing TFEB-mediated lysosomal biogenesis and markedly increased lysosome numbers per cell. This enhanced lysosomal sequestration of chemotherapeutics, away from their intracellular targets, provoked cancer multidrug resistance. However, little is known regarding the fate of lysosome-sequestered drugs. While we suggested that sequestered drugs might be expelled from cancer cells via lysosomal exocytosis, no actual drug-induced lysosomal exocytosis was demonstrated. By following the subcellular localization of lysosomes during exposure to lysosomotropic chemotherapeutics, we herein demonstrate that lysosomal drug accumulation results in translocation of lysosomes from the perinuclear zone towards the plasma membrane via movement on microtubule tracks. Furthermore, following translocation to the plasma membrane in drug-treated cells, lysosomes fused with the plasma membrane and released their cargo to the extracellular milieu, as also evidenced by increased levels of the lysosomal enzyme cathepsin D in the extracellular milieu. These findings suggest that lysosomal exocytosis of chemotherapeutic drug-loaded lysosomes is a crucial component of lysosome-mediated cancer multidrug resistance. We further argue that drug-induced lysosomal exocytosis bears important implications on tumor progression, as several lysosomal enzymes were found to play a key role in tumor cell invasion, angiogenesis and metastasis.

## INTRODUCTION

Lysosomes are acidic intracellular organelles found in all eukaryotic cells, excluding erythrocytes. Lysosomes contain an assortment of hydrolases with optimal catalytic activity at acidic pH; these enzymes breakdown various macromolecules and damaged organelles, arriving at the lysosome both from extracellular milieu, via endocytosis and phagocytosis, as well as from the intracellular compartment also via autophagy [1]. Apart from their role as the major recycling center of the cell, lysosomes are known to partake in a variety of cellular processes including nutrient sensing [1–3], plasma membrane repair [4] and apoptosis [5]. Lysosomal biogenesis is tightly regulated by the master transcriptional regulator E basic helix-loop-helix protein 35 (transcription factor EB, TFEB); TFEB is phosphorylated by mammalian target of rapamycin complex 1 (mTORC1) on serine 211, mediating its retention in the cytoplasm in an inactive state, via binding of phosphorylated TFEB to 14-3-3 proteins [6]. Whereas, activation of TFEB is mediated by release of Ca^2+^ from the lysosome through mucolipin 1 (MCOLN1), bringing about the activation of the Ca^2+^-dependent serine/threonine phosphatase calcineurin, resulting in dephosphorylation of TFEB by calcineurin. As unphosphorylated TFEB cannot bind 14-3-3 proteins, it is translocated from the cytoplasm into the nucleus [7]. Nuclear TFEB activates genes of the coordinated lysosomal expression and regulation (CLEAR) network, responsible for the biogenesis of new lysosomes [8].

Hydrophobic weak base anticancer drugs are known to accumulate at very high levels in lysosomes via cation trapping; these hydrophobic drugs move freely across phospholipid membranes, including the plasma membrane and the lysosomal membrane. Once these drugs encounter the acidic pH of the lysosomal lumen, they become protonated, due to their weak base nature, and thus are no longer able to traverse the lysosomal membrane and become entrapped within the lysosome [9–12]. We have recently demonstrated that lysosomal accumulation of chemotherapeutics mediates drug resistance by sequestering these anticancer drugs in the lysosomes, away from their cellular target sites [11, 13, 14]. We have further shown that the number of drug-accumulating lysosomes per cell is directly correlated with the extent of cellular resistance to the cytotoxic effect of these drugs [14]. We have further reported that lysosomal accumulation of hydrophobic weak base drugs triggers the translocation of TFEB from the cytoplasm into the nucleus, resulting in the transcription of genes from the CLEAR network, and a significant increase in lysosome number per cell [14]. We have thus proposed a model for drug-induced lysosome-mediated drug resistance, in which accumulation of drugs within lysosomes triggers TFEB-mediated lysosomal biogenesis, increasing the number of lysosomes per cell, thus enabling an enhanced lysosomal sequestration of hydrophobic weak base anticancer drugs [14].

Lysosomal exocytosis is a Ca^2+^-dependent process in which lysosomes fuse with the plasma membrane, and release their cargo into the extracellular milieu [1, 15]. In the first step of lysosomal exocytosis, which is Ca^2+^-independent, lysosomes are recruited to a region proximal to the plasma membrane via association with, and movement on microtubules; in the second step which is Ca^2+^-dependent, lysosomes fuse with the plasma membrane, resulting in the release of the lysosomal content into the extracellular compartment [1, 15–18]. Lysosomal exocytosis was found to partake in various physiological processes, including plasma membrane repair [4], large particle endocytosis by macrophages [19], degradation and regeneration of nerve cells [20]. Intriguingly, just like lysosomal biogenesis, lysosomal exocytosis was recently found to be regulated by TFEB; overexpression of TFEB was found to both increase the number of lysosomes near the plasma membrane and induce fusion of these lysosomes with the plasma membrane, which is mediated by Ca^2+^ release from the lysosome [17]. It was recently suggested that lysosomal exocytosis might partake in the clearance of anticancer drugs sequestered in lysosomes, thus possibly contributing to drug resistance in cancer cells, but thus far little was known regarding the correlation between lysosomal drug accumulation and lysosomal exocytosis [10, 21]. In this regard, it is noteworthy that lysosomal exocytosis was shown to be triggered in mouse macrophages by lysosomal alkalinization via treatment with H^+^-ionophores, lysosomal accumulation of weak amines or inhibition of the vacuolar-type H^+^-ATPase (V-ATPase), which is physiologically responsible for the acidification of lysosomes [22]. Given the fact that lysosomal accumulation of hydrophobic weak base chemotherapeutics also results in lysosomal alkalinization [23], it seems likely that it might also induce lysosomal exocytosis.

In addition to a possible role in drug excretion and resistance, lysosomal exocytosis is also likely to play a major role in tumor invasion, metastasis and hence progression [24, 25]; Overexpression and secretion of several lysosomal enzymes including cathepsins B, D, K, and L was found to influence various characteristics of tumor progression, including tumor growth, invasion and angiogenesis [25, 26]. On the other hand, leakage of lysosomal hydrolases including cathepsin D, from the lysosome into the cytosol was found to induce apoptosis or non-apoptotic lysosomal cell death [27], suggesting that exocytosis of damaged lysosomes might also contribute to protection of the cell from release of lysosomal enzymes into the cytoplasm and subsequent cell death.

We undertook the current study to determine whether or not exposure of cancer cells to hydrophobic weak base chemotherapeutics, known to markedly accumulate in lysosomes, alters the intracellular distribution of lysosomes, and induces fusion of lysosomes with the plasma membrane. We further sought to study the impact of lysosomal accumulation of anticancer drugs on the secretion of lysosomal enzymes into the extracellular milieu via lysosomal exocytosis.

## RESULTS

### Lysosomal accumulation of anticancer drugs leads to translocation of lysosomes from the perinuclear zone towards the plasma membrane

**Figure 1:**
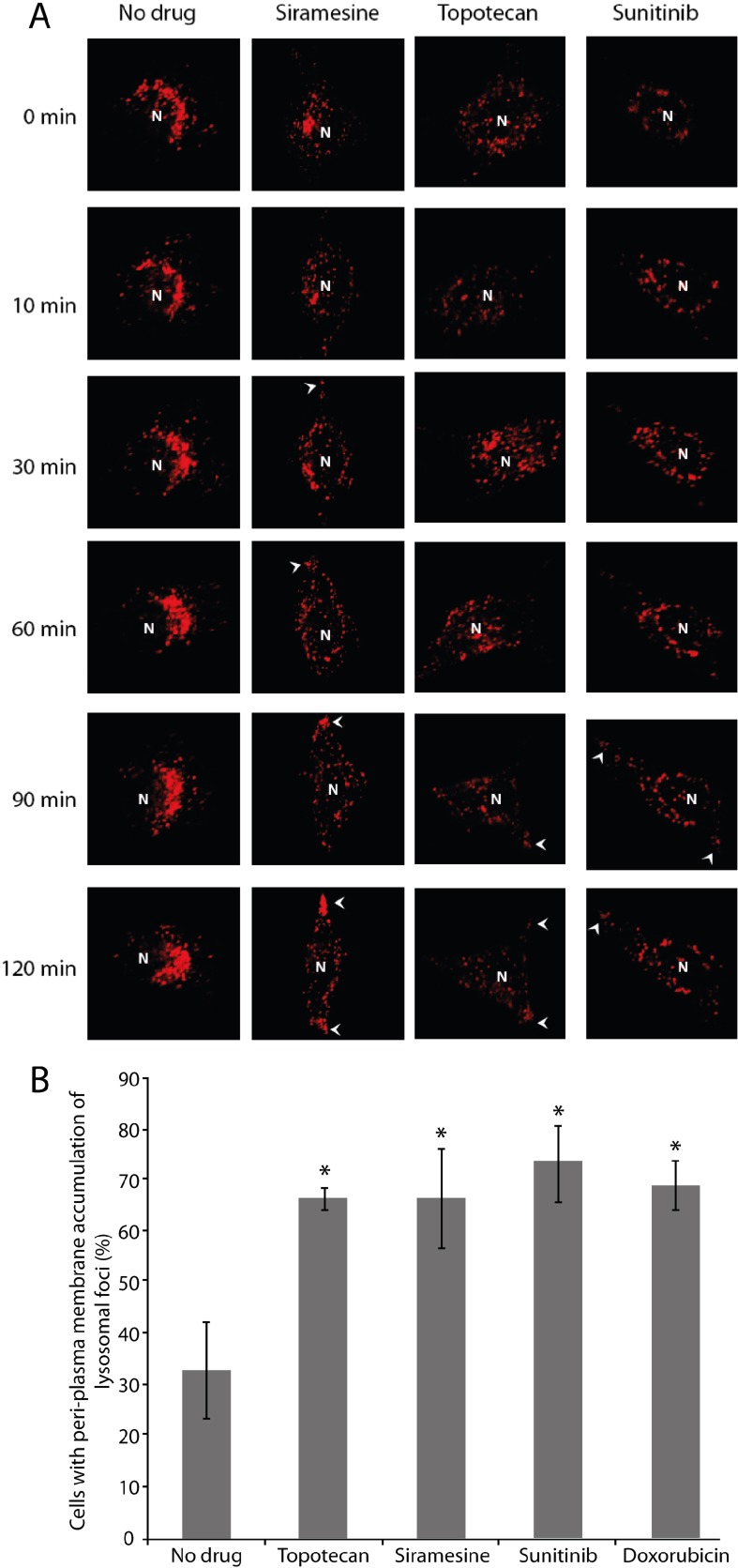
Drug-induced lysosomal translocation towards the plasma membrane HeLa cells stably transfected with LAMP1-mCherry, were treated with siramesine (10 μM), topotecan (10 μM) or sunitinib (10 μM) for 2 hr. Fluorescence microscopy analysis was performed using an inverted confocal microscope (Zeiss LSM 710). Lysosomal foci at the plasma membrane are marked with arrowheads (**A**). Formation of lysosomal foci after 2 hr of treatment with the above drugs and doxorubicin (10 μM) was quantified from 40 fields taken from each sample using a fluorescence microscope InCell analyzer 2000. (**B**) *1-tailed student's *t*-test; *p*-value < 0.05.

It is well established that the majority of mature lysosomes reside in the peri-nuclear zone [28]. Thus, the first step in the process of lysosomal exocytosis is the directional translocation of lysosomes from this peri-nuclear zone towards a location adjacent to the plasma membrane. To determine the possible impact of hydrophobic weak base chemotherapeutics, which are known to highly accumulate in lysosomes (Table 1), on lysosomal localization, as well as translocation of drug-accumulated lysosomes towards the plasma membrane, HeLa cells were stably transfected with lysosomal-associated membrane protein 1 tagged with the fluorescent protein mCherry (LAMP-1-mCherry). These LAMP1-mCherry stably expressing HeLa cells were exposed to a single dose of the topoisomerase I inhibitor topotecan (10 μM) or the receptor tyrosine kinase (RTK) inhibitor sunitinib (10 μM), both of which are hydrophobic weak base anticancer drugs (Log *P* = 0.8, calculated pKa = 9.83 as well as log *P* = 5.2 and pKa = 8.95, respectively; derived from DrugBank [29]), and were previously shown to markedly accumulate in lysosomes [13, 14, 30]. Cells were also exposed to a single dose of siramesine (10 μM), a sigma-2 receptor ligand, which was shown to induce lysosomal membrane permeabilization (LMP) leading to cell death; hence, siramesine was suggested as a novel anticancer compound that targets lysosomes in cancer cells [31, 32]. Upon exposure of cancer cells to these lysosomotropic drugs, subcellular localization of lysosomes was followed by time-lapse confocal microscopy (Figure 1A). Lysosomes were initially localized to the perinuclear zone in all cells analyzed. Control cancer cells which were not exposed to any drug treatment, retained this original perinuclear lysosomal localization throughout the time-course of the experiment. In contrast, cancer cells exposed to the above lysosomotropic anticancer drugs, as well as cancer cells exposed to siramesine, displayed lysosomal translocation towards the plasma membrane, and formation of lysosome foci near the plasma membrane, as early as 30–90 min after initial drug exposure. It is noteworthy that formation of lysosomal foci near the plasma membrane in cells treated with siramesine was more rapid compared to cells treated with topotecan or sunitinib, suggesting that siramesine has a stronger and more immediate effect on lysosome integrity. Indeed, siramesine was previously shown to highly accumulate in lysosomes, and rapidly induce LMP [32]. These findings are in agreement with our results demonstrating that siramesine is the most potent and rapid compound to trigger translocation of lysosomes to the plasma membrane. We hence undertook a quantification of lysosome foci formation near the plasma membrane following 2 hr of exposure to the above drugs, as well as to the topoisomerase II inhibitor doxorubicin (10 μM); it should be noted that the latter is also a hydrophobic weak base (log *P* = 1.27, calculated pKa = 8.94), and was also found to accumulate in lysosomes [33]. This quantification revealed a significant increase in the formation of lysosome foci near the plasma membrane after treatment with these lysosomotropic drugs (Figure 1B).

**Table 1:** LogP and pKa values and molecular structures of the lysosomotropic drugs used in this work

Drug	p*K*a	Log *P*	Structure
Doxorubicin	8.94	1.27	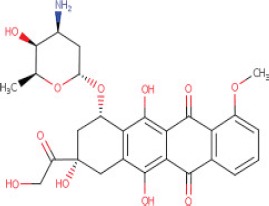
Sunitinib	8.95	5.2	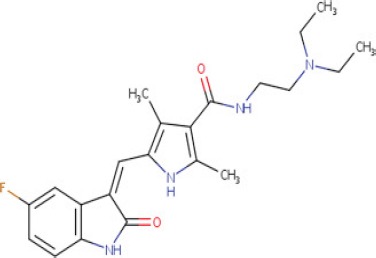
Topotecan	9.83	0.8	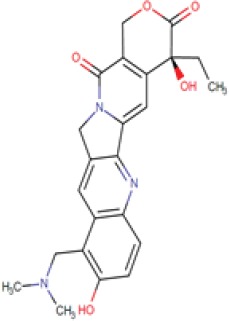
Siramesine	9	8.5	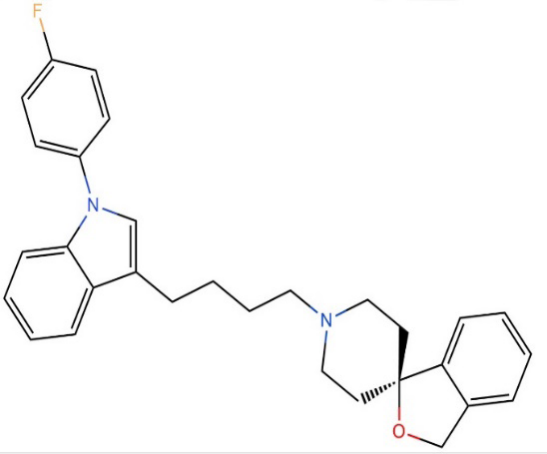

The LogP and pKa values and molecular structure of doxorubicin, sunitinib and topotecan were adapted from the DrugBank database. The LogP and pKa values for siramesine were described by Zimmermann A. et al. [67].

### Lysosomes associate with microtubules and undergo translocation towards the plasma membrane upon exposure to lysosomotropic drugs

**Figure 2:**
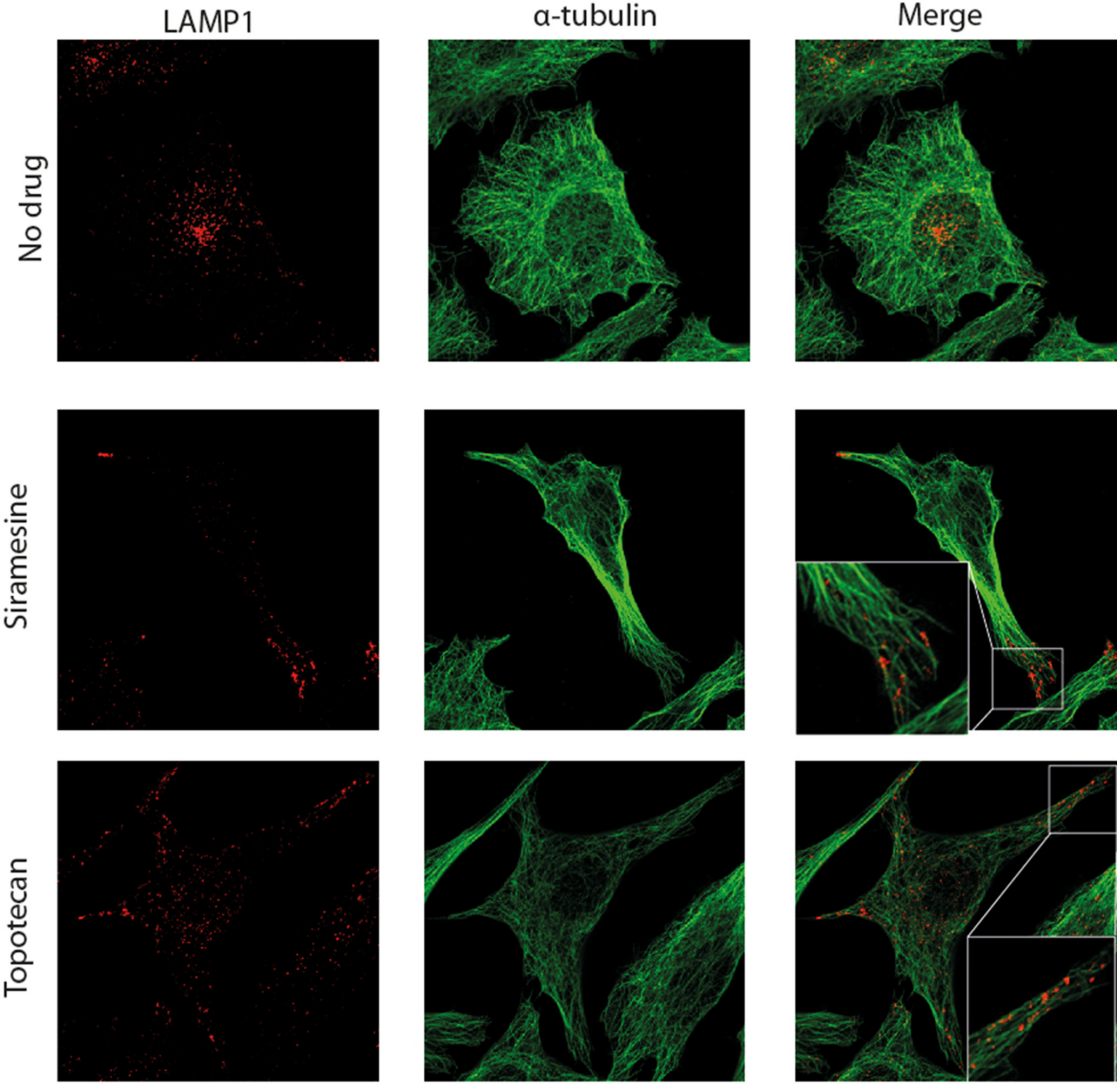
Lysosomes associate with microtubules and translocate towards the plasma membrane HeLa cells were treated with siramesine (10 μM) or topotecan (10 μM) for 1 hr. Immunofluorescence staining was performed using LAMP1 (red)- and α-tubulin (green)-specific antibodies. Fluorescence microscopy analysis was performed using an inverted confocal microscope (Zeiss LSM 710).

**Figure 3:**
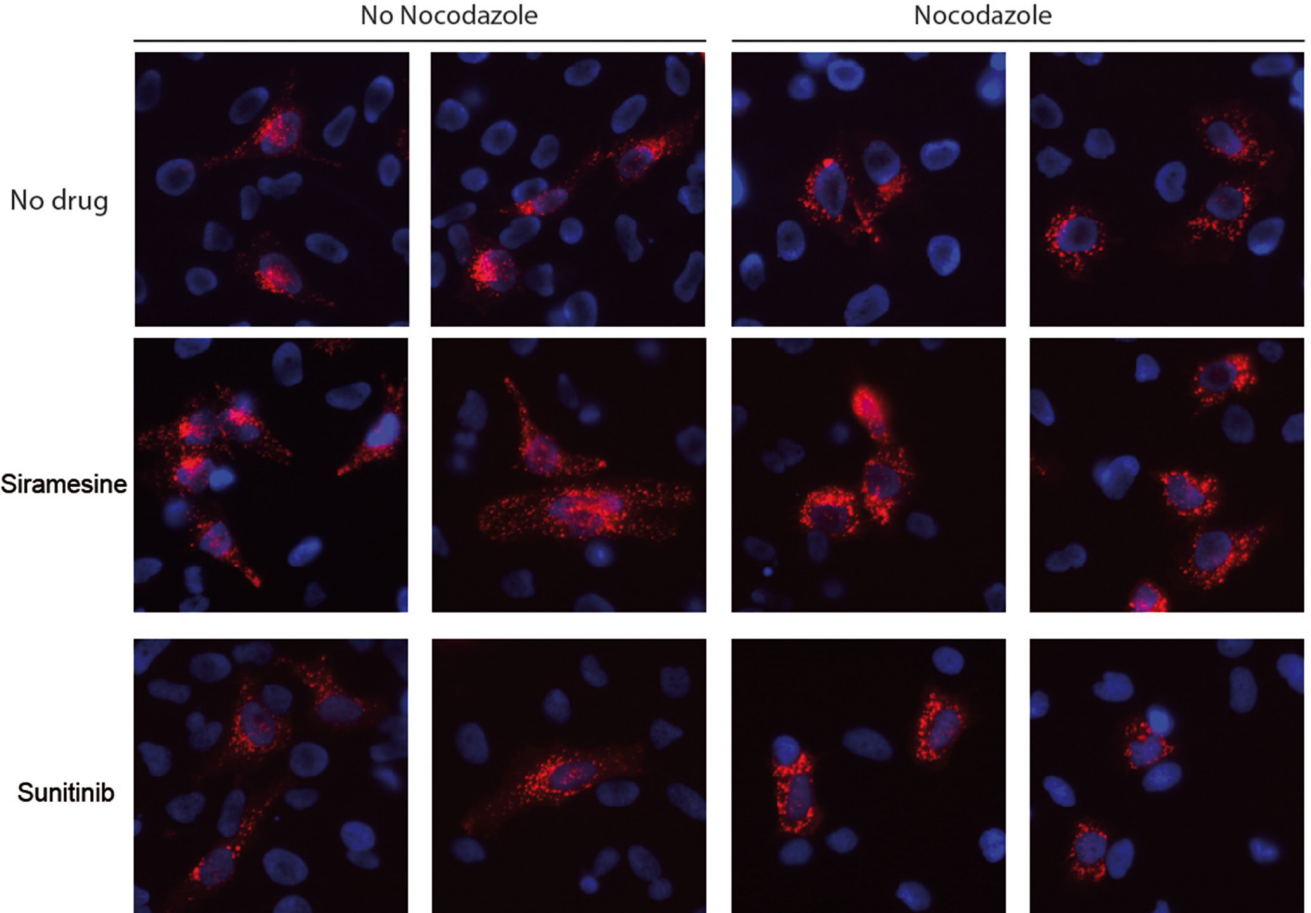
Disruption of microtubules prevents drug-induced lysosomal translocation HeLa cells stably transfected with LAMP1-mCherry (red), were treated with siramesine (10 μM) for 1 hr, with or without pre-treatment with the microtubule disrupting agent nocodazole (1 μM) for 3 hr. Nuclei were stained with hoechst 33342. Fluorescence was followed with Zeiss inverted Cell-Observer microscope.

Long range intracellular movement of lysosomes as well as lysosomal exocytosis, were both shown to depend on microtubules [34, 35]. Having demonstrated drug-induced lysosomal translocation from a peri-nuclear location towards the plasma membrane, we further aimed to determine whether or not this lysosomal translocation is based on association with microtubules. Towards this end, HeLa cells were pulse-treated with siramesine (10 μM) or topotecan (10 μM) for 1 hr, followed by cell fixation and immunofluorescence staining with LAMP1- and α-tubulin-specific antibodies (Figure 2). Expectedly, in untreated cells, the majority of lysosomes were found to reside near the nucleus, and no association of lysosomes with microtubules was found in the periphery of these cells. In contrast, in cells treated with siramesine or topotecan, there was a significant accumulation of lysosomes near the plasma membrane, in agreement with the results described above. In this regard, it is noteworthy that this finding suggests that drug-induced lysosomal translocation is independent of LAMP1 overexpression, which was used in the time-lapse microscopy but not in this experiment. In addition to the translocation of lysosomes towards the plasma membrane in drug-treated cancer cells, we also detected a marked association of lysosomes with microtubules in the extensions leading towards lysosome foci forming near the plasma membrane. These results suggest that drug-induced lysosomal movement towards the plasma membrane is mediated by microtubules. We thus postulated that disruption of microtubule polymerization may abolish this lysosomal translocation. To test this hypothesis, HeLa cells stably transfected with LAMP1-mCherry, were treated with siramesine (10 μM) for 1hr with or without a 3 hr pre-treatment with the microtubule-disrupting agent, nocodazole (1 μM). While control cells which were not exposed to nocodazole formed lysosome foci near the plasma membrane, as described in the previous chapter, disruption of microtubules in cells treated with nocodazole resulted in the retention of lysosomes in the perinuclear zone of cells treated with siramesine (Figure 3). This result demonstrates that intact microtubules are required for drug-induced translocation of lysosomes towards the plasma membrane.

### Accumulation of anticancer drugs in lysosomes leads to fusion of lysosomes with the plasma membrane

**Figure 4:**
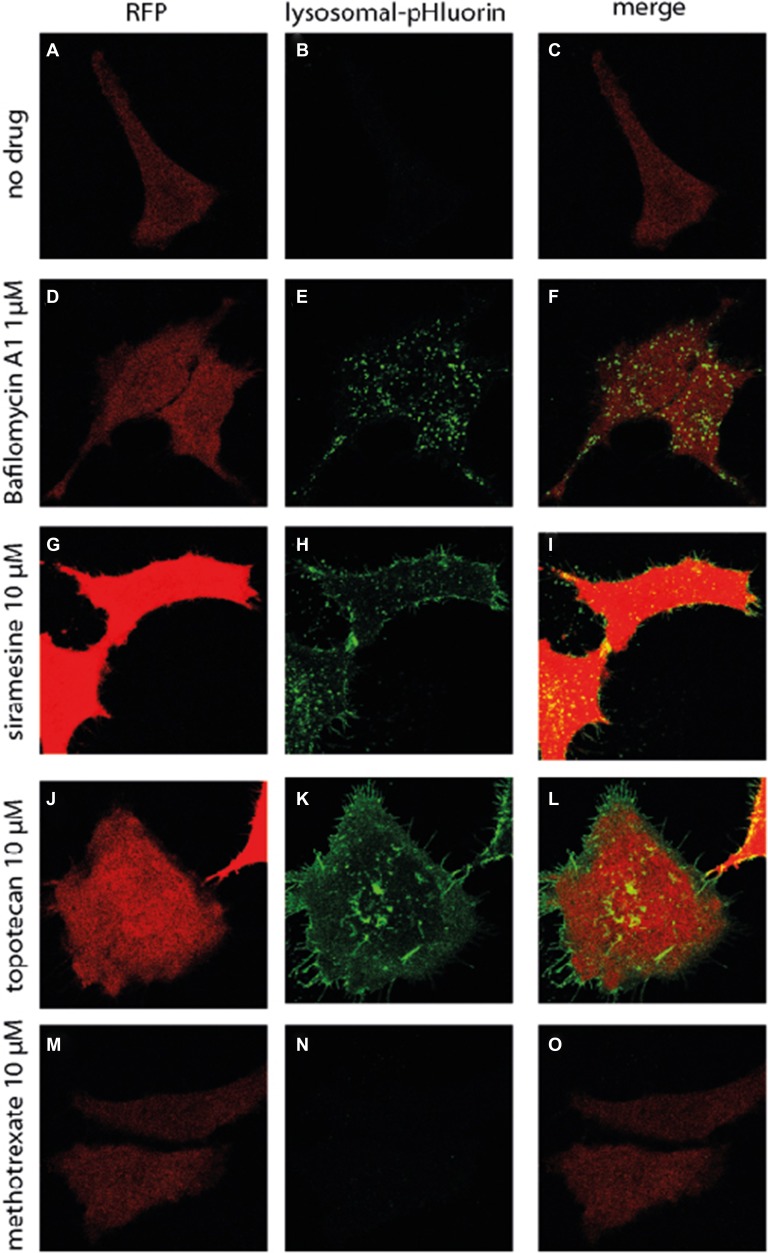
Drug-induced fusion of lysosomes with the plasma membrane HeLa cells were transiently transfected with lysosomal pHluorin (green at neutral pH) and co-transfected with RFP (red) to identify transfected cells and mark cell boundaries. Cells were treated with bafilomycin A1 (1 μM), siramesine (10 μM), topotecan (10 μM) or methotrexate (10 μM) for 2 hr. Fluorescence microscopy analysis was performed using an inverted confocal microscope (Zeiss LSM 710).

Following microtubule-mediated recruitment of lysosomes to the plasma membrane, the second obligatory step of lysosomal exocytosis is the fusion of the lysosomal membrane with the plasma membrane, resulting in the release of the lysosomal cargo into the extracellular compartment [1]. We next aimed to determine whether or not accumulation of anticancer drugs in lysosomes results in the fusion of lysosomes with the plasma membrane; HeLa cells were transiently transfected with lysosomal pHluorin (Lyso-pHluorin). Lyso-pHluorin is a fusion protein between the lysosomal membrane protein CD63 and the green fluorescent protein (GFP) analogue, pHluorin [36]. Similar to GFP, pHluorin has green fluorescence at neutral pH, however under the acidic pH conditions residing in the lysosomal lumen, pHluorin fluorescence is quenched [37]. In Lyso-pHluorin, the pHluorin is facing the lysosomal lumen, and as long as the lysosome remains acidic no pHluorin fluorescence is detected (Figure 4A–4C). In contrast, upon exposure of Lyso-pHluorin-stained HeLa cells to the vesicular H^+^-ATPase (V-ATPase) inhibitor bafilomycin A1 (1 μM, 2 hr), an established lysosome alkalinizing agent, pHluorin fluorescence was detected in the lysosomes, due to the unquenching of pHluorin in the neutralized lysosomal pH (Figure 4D–4F). Little Lyso-pHluorin fluorescence was found on the plasma membrane of bafilomycin A1-treated cells, suggesting that lysosomal alkalinization is not sufficient to induce lysosomal exocytosis. In contrast, Lyso-pHluorin-stained HeLa cells treated with siramesine (10 μM, 2 hr) (Figure 4G–4I) or topotecan (10 μM, 2 hr) (Figure 4J–4L) displayed Lyso-pHluorin fluorescence in both lysosomes, and on the plasma membrane. The alkalinization of lysosomes by these lysosomotropic drugs can be explained either by the accumulation of a high concentration of the weak base drugs in the lysosomal lumen [23], or by LMP, a known effect of siramesine [5], resulting in the disruption of the pH gradient between the lysosome and the cytosol. The gain of Lyso-pHluorin fluorescence at the plasma membrane indicates that siramesine and topotecan induced fusion of lysosomes with the plasma membrane. Specifically, this fusion of lysosomes with the plasma membrane causes the pHluorin to face the extracellular milieu, resulting in the unquenching of pHluorin. As a control of a non-lysosomotropic drug, Lyso-pHluorin-stained HeLa cells were treated with the dihydrofolate reductase (DHFR) inhibitor methotrexate (10 μM, 2 hr), a hydrophilic antifolate anticancer drug which does not accumulate in lysosomes. Expectedly, no pHluorin fluorescence was detected in lysosomes nor on the plasma membrane (Figure 4M–4O), indicating that the reported lysosomal alkalinization and fusion with the plasma membrane is mediated by the lysosomal sequestration of hydrophobic weak base chemotherapeutics but not by hydrophilic drugs which do not accumulate in lysosomes.

### Drug-induced lysosomal exocytosis results in the secretion of the lysosomal protease cathepsin D

**Figure 5:**
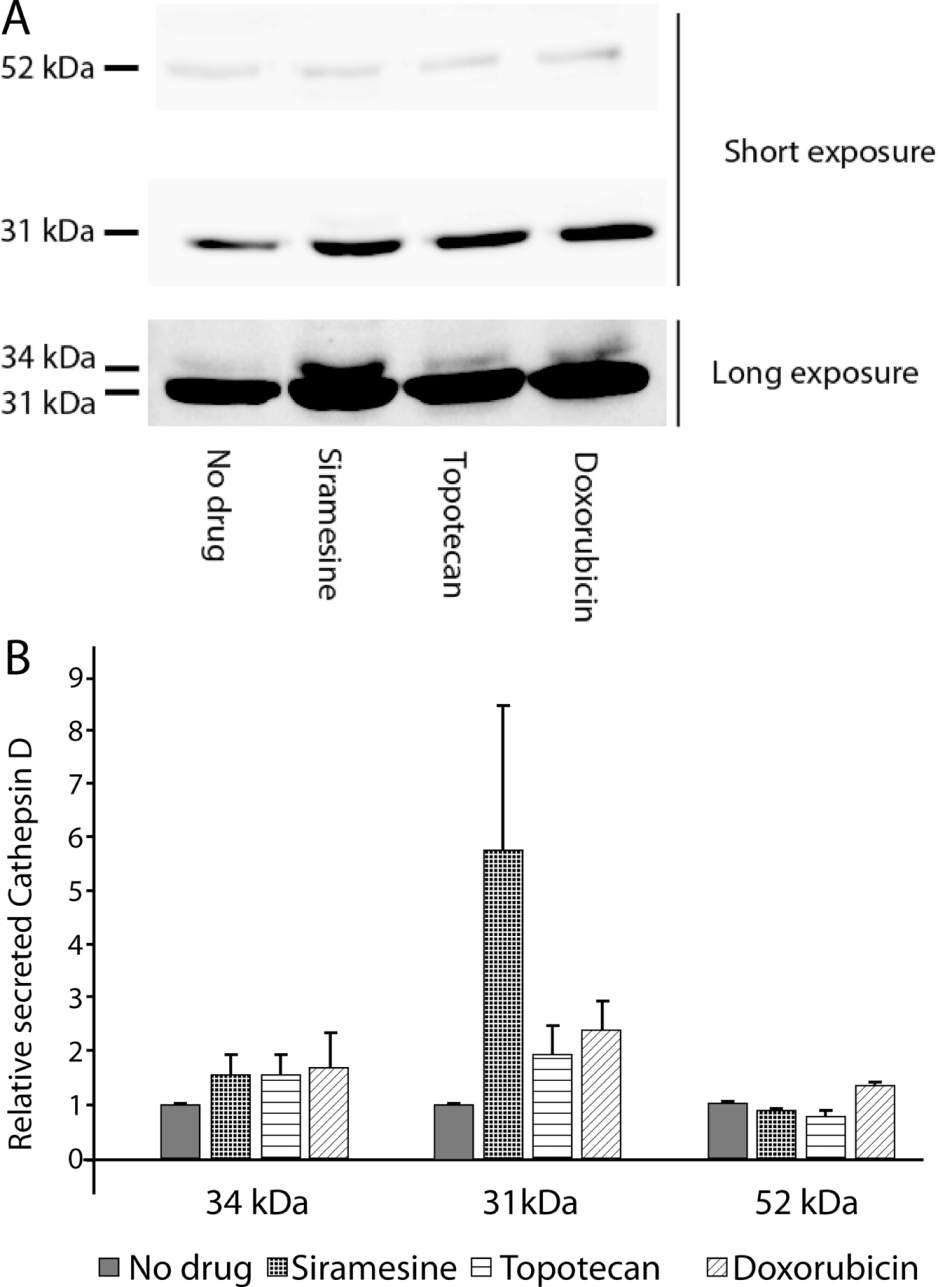
Drug-induced lysosomal exocytosis results in the release of cathepsin D into the medium HeLa cells were treated with siramesine (10 μM), topotecan (10 μM) or doxorubicin (10 μM) for 4 hr. Pro-Cathepsin D (52 kDa) and mature cathepsin D heavy chain (31–34 kDa) levels were determined using Western blot analysis (**A**) and quantified using ImageJ software (**B**). Long and short exposure refer to camera time exposure when photographing the same Western blot membrane. This was performed in order to demonstrate all relevant bands.

Fusion of the lysosome membrane with the plasma membrane culminates in the release of the lysosomal content into the extracellular milieu. To address this lysosomal exocytosis, HeLa cells were exposed to siramesine (10 μM), topotecan (10 μM) or doxorubicin (10 μM) for 4 hr. The content of the lysosomal protease cathepsin D (cath-D) in the extracellular medium was determined by Western blot analysis (Figure 5). Cath-D is initially synthetized on ribosomes in the rough endoplasmic reticulum (RER) as a pre-pro-enzyme, which is then processed in the ER to a 52 kDa pro-cath-D, which is targeted to the lysosome. In the lysosome several additional proteolytic processing steps result in the formation of a 48 kDa intermediate of cath-D, which is further processed to form the 31–34 kDa heavy chain and a 14 kDa light chain of mature cath-D [38, 39]. While the amount of 52 kDa pro-cath-D secreted from the cells did not vary significantly following drug treatment, there was a significant increase in the secretion of the 31 kDa and the 34 kDa mature heavy chain species of cath-D after treatment with all three lysosomotropic drugs (Figure 5). This result indicates that while drug treatment did not affect secretion of the unprocessed pro-cath-D directly from the trans-Golgi, it did induce release of the mature cath-D found exclusively in lysosomes, suggesting that drug-induced secretion of lysosomal enzymes is mediated by lysosomal exocytosis, and not by failure to target lysosomal enzymes from the Golgi to the lysosome. Thus, this finding shows that lysosomal accumulation of anticancer drugs results in an enhanced secretion of the lysosome content, including lysosomal enzymes, into the extracellular milieu via lysosomal exocytosis.

## DISCUSSION

**Figure 6:**
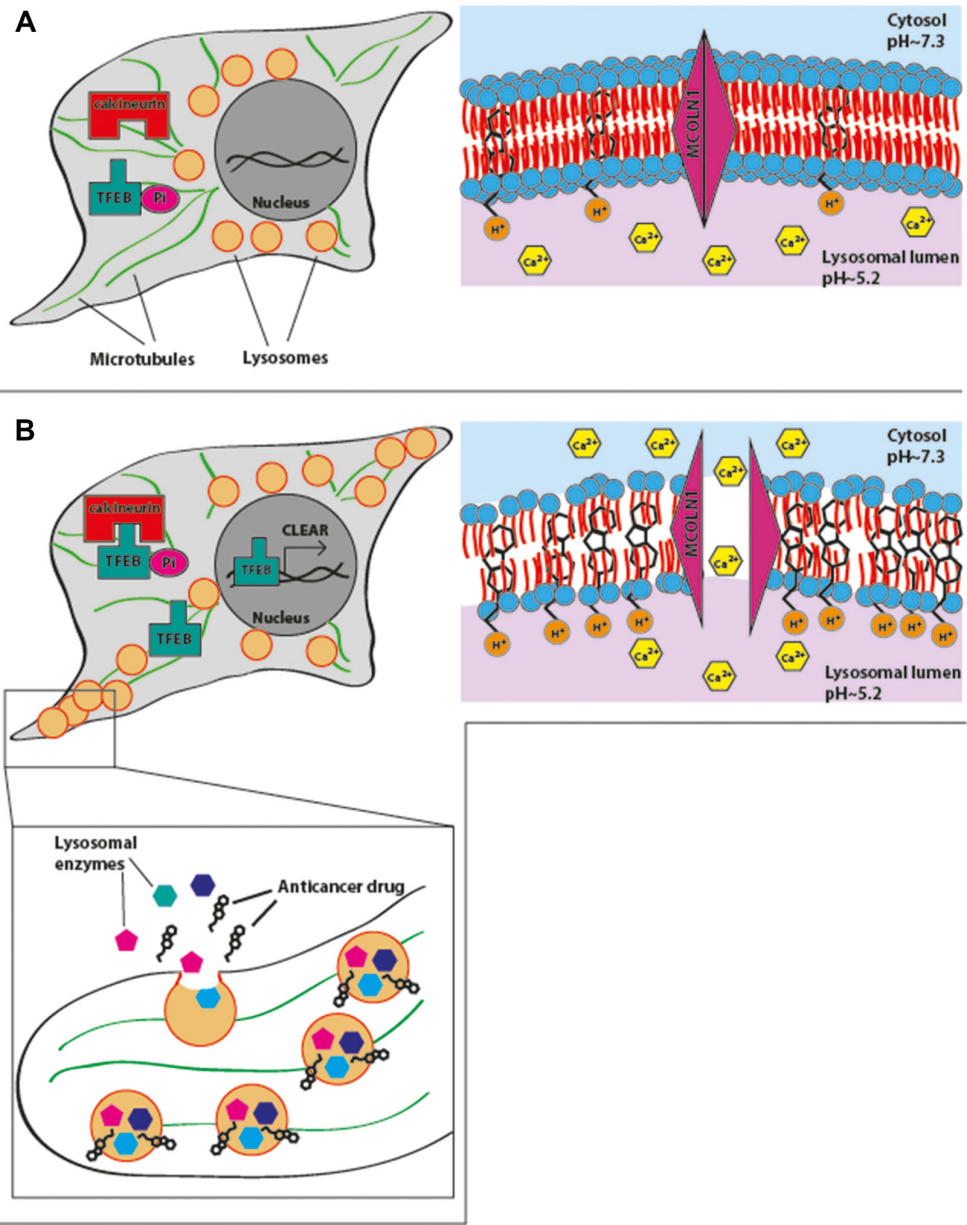
A summary model for drug-induced lysosomal exocytosis In the absence of lysosomal stress, lysosomes are located near the nucleus. Hydrophobic weak base chemotherapeutics accumulate in the lysosomal membrane, due to protonation of basic residues facing the acidic lumen of lysosomes and retention of hydrophobic residues in the lipid bilayer (**A**). Accumulation of high concentration of drugs in the lysosomal membrane induces lysosomal membrane fluidization and permeabilization, resulting in the release of Ca^2+^ ions from the lysosome into the cytoplasm. Release of Ca^2+^ activates calcineurin which dephosphorylates TFEB, resulting in its translocation into the nucleus and activation of the CLEAR pathway. Consequently, lysosomes associate with microtubules and are translocated towards the plasma membrane. Lysosomes fuse with the plasma membrane and release their content, including sequestered drugs and lysosomal enzymes, into the extracellular milieu (**B**).

Herein we have demonstrated that exposure of cancer cells to hydrophobic weak base chemotherapeutics which highly accumulate in lysosomes via cation-trapping, results in a rapid onset of lysosomal exocytosis, which is initiated by microtubule-mediated translocation of lysosomes from the perinuclear zone (Figure 6A) towards the plasma membrane (Figure 6B). This resulted in the formation of lysosome foci in the periphery of the cell, followed by fusion of the lysosome membrane with the plasma membrane and consequent release of the lysosomal cargo to the extracellular milieu (Figure 6B). Thus, the present study constitutes the first evidence of anticancer drug-induced lysosomal exocytosis.

We therefore propose a summary model (Figure 6) which is based upon our present findings as well as on those obtained by other research groups, in order to illustrate the putative sequence of molecular events occurring upon exposure of cancer cells to lysosomotropic cytotoxic agents, culminating in lysosomal biogenesis, microtubule-dependent translocation of perinuclear lysosomes to the plasma membrane, lysosome-plasma membrane fusion and finally exocytosis. As previously shown, weakly basic hydrophobic anticancer drugs represented by doxorubicin, mitoxantrone and topotecan are first intercalated into the external hemilayer of the plasma membrane and presumably undergo a relatively rapid flip-flop to the inner leaflet of the plasma membrane [40]. Upon internalization, they may also undergo rapid incorporation into the external leaflet of the lysosome membrane followed by flip-flop into the inner membrane leaflet (Figure 6A). This intercalation into the hydrocarbon lipid core of the lysosomal membrane occurs via the insertion of the multi-aromatic ring structure of these anticancer drugs, for example via the planar hydrophobic anthracene structure of doxorubicin and mitoxantrone. Hence, once the amino residue of the daunoseamine group of these anthracyclines (Table 1) faces the highly acidic lumen of the lysosome (pH = 5.2), it rapidly undergoes protonation rendering them cationic compounds (Figure 6A). Consequently, these cationic anticancer drugs are entrapped in lysosomes and are no longer able to diffuse out across the lysosomal membrane. This results in their accumulation at very high levels in the lysosomal membrane. As we have previously shown, these high levels of amphiphilic molecules in biological membranes result in a marked alteration in membrane fluidity (i.e. membrane fluidization) [41] provoking LMP due to a detergent-like effect on the lysosomal membrane. Consequently, this LMP is also accompanied by leakage of Ca^2+^ ions from the lysosome into the cytosol via mucolipin 1, which presumably assumes an open Ca^2+^ channel configuration. This efflux of Ca^2+^ ions, activates dormant calcineurin which resides in the cytoplasm (Figure 6B). The latter which is now endowed with a Serine/Threonine phosphatase activity, dephosphorylates TFEB at residue 211 [17], resulting in translocation of TFEB to the nucleus. Nuclear TFEB orchestrates the transactivation of the CLEAR pathway, hence inducing lysosomal biogenesis and exocytosis. However, it should be noted that at least another group of lysosomotropic compounds was found to target lysosomes and result in LMP and/or lysosomal cancer cell death via mechanisms distinct from those described above [31, 42]; this group of compounds is represented by siramesine. In one mode of action, siramesine was found to exhibit high-affinity binding to phosphatidic acid and to acidic phospholipids [42]. Thus, upon entrapment in lysosomes, lysosomotropic compounds appear to inflict various deleterious effects to the membrane of the lysosomes, provoking lysosomal biogenesis and exocytosis.

The first implication of our finding of drug-induced lysosomal exocytosis is the release of the anticancer drug cargo that highly accumulated in lysosomes. Thus, this constitutes a novel mechanism of multidrug resistance based on drug sequestration and efflux in addition to any co-existing multidrug efflux transporter activity [12]. We have previously shown that lysosomal sequestration of anticancer drugs is a mechanism of resistance of cancer cells to hydrophobic weak base anticancer drugs [10, 14]. This novel mode of resistance is based on lysosomal sequestration of chemotherapeutic drugs in lysosomes, hence preventing them from reaching their intracellular target sites, abolishing their cytotoxic activity [10, 14]. While accumulation of anticancer drugs within lysosomes, occurs predominantly via ion-trapping, active transport of certain drugs into lysosomes was also described [10, 43]; however, little was known regarding the fate of the sequestered drugs or the drug-loaded lysosomes. Our current findings demonstrate that drugs sequestered in lysosomes do not remain entrapped within these lysosomes indefinitely, and are extruded from the cell via lysosomal exocytosis. We thus postulate that lysosome-mediated drug resistance is a two-step process: in the first step, drugs are sequestered in lysosomes away from their intracellular target sites, and in the second step they are extruded out of the cell via lysosomal exocytosis. Both lysosomal biogenesis and exocytosis, which we have previously shown to be induced by lysosomal accumulation of anticancer drugs [14], are mediated by the release of Ca^2+^ ions from the lysosome, resulting in the activation of calcineurin, a Ca^2+^-dependent serine/threonine phosphatase, with consequent dephosphorylation of TFEB and its translocation to the nucleus [7, 17]. As some lysosomotropic drugs were shown to induce LMP, leading to lysosome-mediated cell death [5], we postulate that Ca^2+^ release from drug-permeabilized lysosomes, and subsequent lysosomal exocytosis, might also serve as a cellular defense mechanism against the cytotoxic release of the lysosomal content into the cell. Thus, these findings suggest that drug-induced lysosomal exocytosis provides a two-tier defense mechanism, both by extrusion of cytotoxic drugs from the cell as well as by preventing LMP-mediated cell death.

The fact that drug-induced activation of TFEB promotes not only lysosomal biogenesis, as previously described [8], but also lysosomal exocytosis, is in agreement with a recent study demonstrating that nuclear localization of TFEB is indeed required for transcriptional activation of lysosomal exocytosis [17]. We thus propose that drug-induced lysosomal biogenesis and lysosomal exocytosis must go hand in hand, in order to maintain the cellular lysosomal capacity needed to sustain the required level of lysosomal activity for cell survival; while lysosomal exocytosis reduces the number of lysosomes per cell due to the fusion of drug-loaded lysosomes with the plasma membrane, lysosomal biogenesis is activated to replace these lysosomes with newly synthetized lysosomes that have not yet accumulated hydrophobic weak base drugs.

As demonstrated herein, drug-induced lysosomal exocytosis results in the secretion of lysosomal enzymes into the extracellular milieu. In this respect, it is important to note that several lysosomal enzymes including cathepsin B, D, K and L, were found to partake in various malignant processes, both within, but also outside the cell, including invasion, metastasis and activation of angiogenesis [25, 26]. Cath-D, which was shown herein to be highly secreted from cancer cells via drug-induced lysosomal exocytosis, was previously found to be overexpressed in breast cancer cell lines and in most breast cancers [27]. Furthermore, in clinical studies which examined cath-D levels in primary breast cancer tumors, high levels of cath-D were shown to be a poor prognostic marker, as they correlated with enhanced metastasis and shorter survival [27, 44]. Cath-D was also found to be highly secreted from breast and colorectal cancer cells, where it contributed to both the invasive and metastatic potential of these cells [45]. While thus far cath-D was mainly found to be secreted from cancer cells in its catalytically inactive pro-enzyme, 52 kDa form, it was suggested that it can be activated in the acidic microenvironment of the tumor [46]. Intriguingly, it was recently demonstrated that some of the contribution of cath-D to the malignant progression is not dependent on its catalytic activity, as the cath-D mutant^D231N^, which is proteolytically inactive, retained mitogenic activity in cancer cells [47]. One other lysosomal enzyme relevant to tumor progression, among the multiple hydrolytic enzymes present in lysosomes, is the unique enzyme heparanase; it should be noted that the final stage in the activation of heparanase occurs in the lysosome via proteolytic cleavage of a linker region encompassing Ser_110_–Gln_157_ mediated by cathepsin L [48], hence liberating an N-terminal 8-kDa subunit as well as a C-terminal 50-kDa subunit, which remain associated as a noncovalent heterodimer in the mature active heparanase [49]. Thus, heparanase, is an endoglucuronidase which cleaves heparan sulfate (HS), resulting in alteration of the structure and function of heparan sulfate proteoglycans (HSPG) as well as tumor-dependent remodeling of both cell surface and the extracellular matrix (ECM) [50–54]. These important activities markedly impact multiple regulatory pathways, predominantly via acceleration of cell invasion, angiogenesis and metastasis also by enhancement of the bioavailability of growth factors and cytokines bound to HS [50, 53]. Heparanase, present in lysosomes and late endosomes, plays an essential housekeeping role in catabolic processing of internalized HSPGs [55], autophagy [56] and exosome formation [57]. Importantly, heparanase can be trafficked to the cell surface or released into the ECM, where it affects breakdown of extracellular pools of HS [58]. Specifically, heparanase-mediated hydrolysis of HS in the ECM has several effects on the behavior of nearby cells. Weakening of structural HS networks in the ECM directly facilitates cell motility and invasion into surrounding tissues [59]. Latent pools of growth factors entrapped within HS are released upon breakdown by heparanase and subsequently promote increased cell proliferation, motility and angiogenesis [60, 61]. HS fragments generated by heparanase activity can also activate downstream signaling cascades [62]. Whereas controlled heparanase activity plays an important role in physiological processing of the ECM, tissue repair [63], hair follicle growth [64] and immune surveillance, aberrant heparanase expression is associated with inflammation and cancer, strongly correlating with metastasis and dismal clinical prognosis [51, 52, 54, 65].

In conclusion, exocytosis of lysosomes sequestering anticancer drugs presents a major advantage in the crucial adaptation to the harsh tumor microenvironment that is both hypoxic, acidic, and nutrient-deprived. Thus, angiogenesis, tumor cell invasion and tumor cell spread to distant sites for colonization in well-nourished and well-perfused healthy tissues, presents a key advantage for malignant tumors which undertake the cellular decision of lysosomal biogenesis and lysosomal exocytosis. In this respect, novel targeting of lysosomal biogenesis and exocytosis may achieve not only the overcoming of chemoresistance but may also readily abolish tumor invasion, angiogenesis, metastasis and acquisition of an aggressive tumor phenotype. Therefore, our present findings bear important implications for the possible development of novel targeted cancer therapeutics.

## MATERIALS AND METHODS

### Chemicals

Siramesine, topotecan, 4′,6′-diamidino-2-phenylindole (DAPI) and hoechst 33342 were obtained from Sigma-Aldrich (St. Louis, MO, USA). Sunitinib was a kind gift from Prof. A.W. Griffioen, VU Medical Center, Amsterdam, The Netherlands. Doxorubicin was from Tocris Bioscience (Bristol, United Kingdom). Bafilomycin A1 was purchased from Enzo Life Sciences (Farmingdale, NY, USA).

### Cell culture and transfections

Human cervical cancer HeLa cells were maintained in RPMI-1640 medium (Gibco, Paisley, UK), supplemented with 10% fetal bovine serum, 2 mM glutamine, 100 μg/ml penicillin and streptomycin (Biological Industries, Beth-Haemek, Israel) in a humid atmosphere containing 5% CO_2_ at 37°C. HeLa cells were transiently transfected using Linear Polyethylenimine (PEI, MW 25,000) transfection reagent (Polysciences, Pennsylvania, USA) at a ratio of 3 μg PEI : 1 μg DNA. For stable transfection with LAMP1-mCherry, 24 hr after transfection, cells were subjected to G-418 selection (400 μg/ml; Sigma-Aldrich, St. Louis, MO, USA) in the growth medium. pLAMP1-mCherry was a gift from Amy Palmer (Addgene plasmid # 45147). pCMV-lyso-pHluorin was a gift from Christian Rosenmund (Addgene plasmid # 70113). pTurbo-RFP-C was from Evrogen (Moscow, Russia).

### Live cell imaging

HeLa cells were plated in 24-well glass bottom plates (*In Vitro* Scientific, CA, USA). For lysosome localization studies and pHluorin experiments, cells were exposed to siramesine (10 μM), topotecan (10 μM), or sunitinib (10 μM) for 2 hr. Fluorescence was followed using an inverted confocal microscope (Zeiss LSM 710). For quantification of lysosomal plasma membrane foci, cells were exposed to 10 μM siramesine, topotecan, doxorubicin or sunitinib for 2 hr, followed by fluorescence microscopy analysis using an InCell analyzer 2000 (GE Healthcare Bio-Sciences, Pittsburgh, PA, USA). To achieve nuclear staining prior to fluorescence imaging, cells were incubated with 2 μg/ml Hoechst 33342 in growth medium for 10 min. At least 200 cells from a minimum of 40 images were analyzed for each treatment. Lysosomal foci near the plasma membrane were identified by an objective juxtaposition of the lysosomes to the plasma membrane, hence being distant from the nucleus.

### Immunofluorescence

HeLa cells were seeded in 24-well plates on sterile glass coverslips and incubated for 24 hr at 37°C. Cells were then treated with 10 μM siramesine or topotecan for 1 hr. Cell fixation, permeabilization and immunofluorescent staining were performed as previously described [14]. LAMP1 was visualized using rabbit anti-LAMP1 polyclonal antibody (ab24170; 1:1000 dilution, Abcam, Cambridge, MA, USA). Microtubules were visualized using anti-α-tubulin mouse monoclonal antibody T9026 (1:500 dilution, Sigma-Aldrich, St. Louis, MO, USA). Nuclei were counterstained with the DNA dye DAPI (0.5 μg/ml).

### Growth medium protein concentration and Western blot analysis

For cathepsin D secretion experiments, HeLa cells were plated in T75 flasks. Prior to the addition of drugs, monolayer cells were washed with PBS to remove growth medium proteins, the full medium described above was replaced by a serum-free RPMI-1640 medium. Cells were then treated with 10 μM siramesine, topotecan or doxorubicin for 4 hr. The medium from each flask was collected and passed through a 0.45 μM filter unit to dispose of detached cells. Proteins in the medium were concentrated using Amicon ultra-15 centrifugal filters (Merck Millipore, Billerica, Massachusetts, USA). Cath-D levels in the medium were determined by Western blot analysis, which was performed as previously described [66], using an anti-cath- D mouse monoclonal antibody (ab6313; 1:1000 dilution, Abcam, Cambridge, MA, USA). Band intensity was quantified using ImageJ software.
